# Polar Cryogenic Impact Behavior of Selective Laser Melted Ti-6Al-4V Alloy: Effects of Scanning Strategies and Notch Orientation

**DOI:** 10.3390/ma18174177

**Published:** 2025-09-05

**Authors:** Hantao Chen, Wenyong Guo, Xiaofeng Li, Xinglong Pan, Jianxiang Zhang, Li Yu, Yan Zeng

**Affiliations:** College of Power Engineering, Naval University of Engineering, Wuhan 430030, China; 1920191077@nue.edu.cn (H.C.); guowy202@163.com (W.G.); 2120213016@nue.edu.cn (X.L.); paxilo619@163.com (X.P.); m23180201@nue.edu.cn (J.Z.)

**Keywords:** Ti-6Al-4V, selective laser melting, polar cryogenic impact behavior, mechanical anisotropy

## Abstract

The anisotropic mechanical properties of selective laser melting (SLM)-processed Ti-6Al-4V (TC4) alloy hinder its deployment in polar marine equipment. This study systematically probes the relationships between laser scanning strategies (unidirectional vs. 67°-rotated scanning between layers), notch orientation (governing loading direction), and cryogenic impact energy of SLM-TC4. Charpy impact tests from −60 °C to 20 °C were performed on V-notched specimens fabricated with distinct scanning strategies and notch orientations (top/side surfaces). The analysis of impact energy data and macro/micro-fractography demonstrates that impact energy declines markedly with decreasing temperature, showing a 25–35% reduction at −60 °C versus 20 °C while exhibiting enhanced data consistency under cryogenic conditions. Notably, specimens fabricated with 67°-rotated scanning between layers achieve higher impact toughness than unidirectionally scanned equivalents. Moreover, for identical scanning strategies, side-notched specimens consistently outperform top-notched specimens, evidencing superior interfacial bonding strength between deposited layers relative to bonding within individual layers. Within individual layers, toughness normal to the laser scan path exceeds that parallel to the path. However, controlling ductile-to-brittle transition behavior and precluding brittle failure are imperative for SLM-TC4 components in polar cryogenic service. This work delivers essential quantitative benchmarks and experimental validation for optimizing SLM processing in critical polar vessel components.

## 1. Introduction

The accelerated melting of Arctic ice and snow facilitates global engagement in Arctic shipping and resource exploitation. Polar vessels, functioning as critical infrastructure, provide essential capabilities for accessing target polar marine zones and conducting operational activities [1]. During navigation in these regions, key components including rudder blades, propellers, pumps and valve assemblies experience frequent impacts from fragmented ice and sustain oscillatory excitation forces. These operational demands impose stringent requirements on the cryogenic impact performance of structural materials in critical systems, necessitating not only adequate strength and toughness at polar low temperatures but also sufficient manufacturability for geometrically complex components [2,3,4].

Titanium alloys, exemplified by Ti-6Al-4V (TC4), demonstrate superior strength-to-mass ratios, elevated tensile strength, exceptional corrosion resistance, and enhanced high-temperature stability compared to conventional alloys, along with advantageous properties including low thermal conductivity, minimal thermal expansion coefficient, and non-magnetic characteristics that enable extensive aerospace and marine applications [5,6,7,8]. By extension, low-strength grades (TA2/TA3/TA4) serve in non-pressure castings, pipelines, valves, and heat exchangers; medium-strength variants (TA18M/TA17/ZTC4) in pressure castings, ship structures, propellers, and hatch covers; while high-strength alloys (TC4/TC4ELI/TC11) are utilized for submarine pressure hulls—as evidenced by China’s *Jiaolong* deep-sea submersible—high-pressure vessels, springs, and fasteners [9]. However, the traditional manufacturing of complex titanium components suffers from low productivity and high energy consumption, elevating production costs for medium/high-strength grades and constraining their deployment. In this context, selective laser melting (SLM) emerges as a rapidly advancing metal additive manufacturing technology capable of meeting customized, high-precision demands for geometrically complex parts [10]. TC4 alloy, being a predominant SLM material due to its balanced properties, holds significant promise for marine applications, with growing vessel integration of additively manufactured components and onboard SLM equipment enabling the emergency repair/replacement of damaged parts [11].

Recent years have witnessed growing scholarly focus on the mechanical properties of titanium alloys under varying temperatures. Sotto et al. [12] conducted tensile, shear, and compression tests across different strain rates and temperatures, developing constitutive models subsequently simulated via LS-DYNA. Lin et al. [13] employed ABAQUS to investigate the cryogenic mechanical behavior and constitutive modeling of TC4 titanium alloy, introducing modifications to the Johnson–Cook model specifically for low-temperature environments. Semenova et al. [14] examined temperature effects on ultrafine-grained (UFG) TC4, revealing negative correlations between temperature and both yield/ultimate tensile strengths, with Charpy-absorbed energy decreasing as temperature declined. Lv et al. [15] fabricated TC4 specimens via laser melting deposition (LMD), performing electro-assisted high-temperature tensile tests at strain rates of 0.001 s^−1^, 0.005 s^−1^, and 0.01 s^−1^ across 650 °C, 700 °C, and 750 °C, demonstrating negative and positive correlations of flow stress with temperature and strain rate, respectively. Qian et al. [16] compared fatigue performance between SLM-produced and forged TC4 specimens, identifying that internal pores and micro-defects in additively manufactured samples reduced fatigue life while increasing data scatter relative to forged counterparts. Ji et al. [17] demonstrated that milling post-SLM processing significantly improved surface integrity of TC4 alloy, reducing roughness Ra and enhancing fatigue life. Wang’s [18] research on SLM process optimization for ultra-cryogenic TC4 components reported room-temperature yield and tensile strengths reaching 1214 MPa and 1255 MPa, respectively—over 20% higher than conventionally rolled TC4—with superior work-hardening capacity and further strength enhancement at cryogenic temperatures.

In summary, current research on titanium alloys predominantly focuses on conventionally manufactured variants under extreme low/high temperatures or ambient conditions, with studies on SLM-TC4 largely concentrated on process optimization rather than mechanical properties—particularly impact behavior in the polar cryogenic regime (−60 °C to 20 °C). Concurrently, the inherent microstructural heterogeneity of SLM-TC4 components induces pronounced mechanical anisotropy [19]. To address these gaps, this work employs Charpy pendulum impact testing across polar-relevant temperatures (−60 °C to 20 °C in 20 °C increments), systematically evaluating SLM-TC4 specimens fabricated with distinct laser scanning strategies (interlayer unidirectional vs. 67°-rotated scanning between layers) and notch orientations (governing loading direction) [20]. Through the integrated analysis of impact energy data and macro/micro-fractography, we elucidate the influences of temperature, scanning strategy, and notch orientation on impact performance, thereby offering practical guidance for additive manufacturing methodology and material selection in critical polar marine structural components.

## 2. Materials and Methods

### 2.1. Test Materials

The raw material for additive manufacturing was TC4 titanium alloy powder, with post-processed impact specimen composition detailed in Table 1.

**Figure 1:**
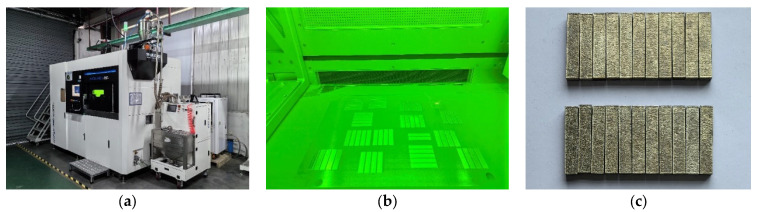
Materials processing: (**a**) FS350-4M system; (**b**) processing procedure; (**c**) as-printed metallic specimens.

SLM fabrication employed an FS350-4M system (Farsoon Technologies, Changsha, China), with key process parameters detailed in Table 2. The system and processing procedures are depicted in Figure 1a,b. Representative as-printed metallic specimens (12 mm × 12 mm × 58 mm, Figure 1c) underwent subsequent machining to standard impact dimensions.

**Figure 2:**
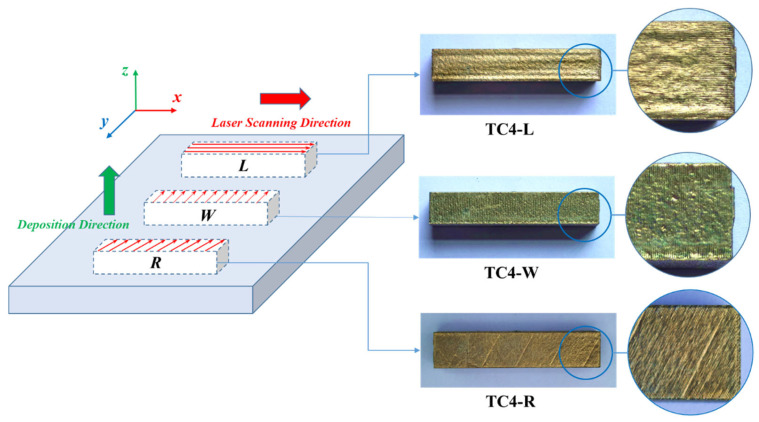
Laser scanning strategies and corresponding specimens.

Specimens were designated according to laser scanning strategies: TC4-L for interlayer unidirectional scanning along the major axis (x-direction, parallel to specimen length), TC4-W for interlayer unidirectional scanning along the minor axis (y-direction, parallel to width), and TC4-R for 67°-rotated scanning between layers, as schematically illustrated in Figure 2.

### 2.2. Specimen Preparation

**Figure 3:**
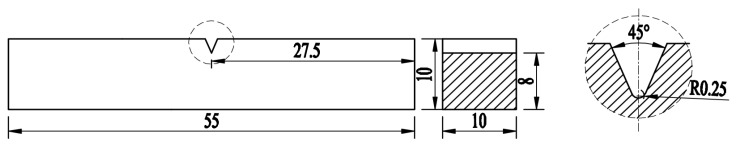
Geometrical configuration and parameters of impact specimen.

Three types of SLM-fabricated TC4 titanium alloy specimens were machined into V-notched configurations according to GB/T 229-2020, with dimensions of 55 mm length and 10 mm × 10 mm square cross-section [21]. The V-notch featured a 2 mm depth and 0.25 mm root radius at the specimen midspan, as detailed in Figure 3.

**Figure 4:**
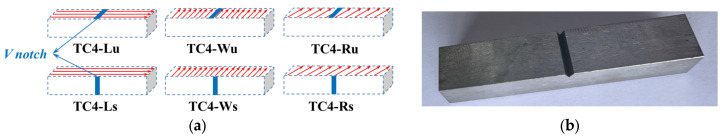
Impact test specimens: (**a**) schematic of V-notch locations; (**b**) physical specimens.

Given the anisotropic mechanical properties of SLM-TC4, V-notches were machined on both top and side surfaces of TC4-L, TC4-W, and TC4-R specimens (Figure 4a) to enable comparative analysis. Physical specimens are shown in Figure 4b. This yielded six specimen groups, subsequently designated TC4-Lu, TC4-Ls, TC4-Wu, TC4-Ws, TC4-Ru, and TC4-Rs.

### 2.3. Charpy Pendulum Impact Test

**Figure 5:**
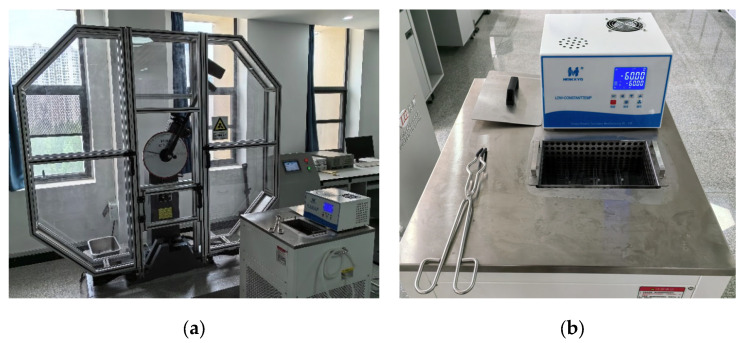
Experimental setup for cryogenic impact testing: (**a**) Charpy pendulum impact tester; (**b**) ethanol-filled cryogenic bath.

The experimental setup is depicted in Figure 5. A JBS-300Z Charpy pendulum impact tester (Shandong Liangong Testing Machine Co., Ltd., Dezhou, China), with a maximum impact energy capacity of 300 J and striker radius of 2.0–2.5 mm, was employed. Given that polar environments routinely reach −60 °C, five test temperatures were selected: 20 °C, 0 °C, −20 °C, −40 °C, and −60 °C. Three replicate tests were conducted at each temperature, achieved by fully immersing specimens in an ethanol-filled cryogenic bath. During testing, specimens were firmly seated against the anvils, ensuring ≤0.5 mm offset between the notch symmetry plane and anvil midplane, with the striker centered opposite the specimen notch.

This protocol provides impact energy values and fracture surfaces for all specimen groups across tested temperatures. Under standardized specimen geometry, the impact energy value directly correlates with toughness behavior, serving as a reliable proxy for evaluating impact characteristics. Subsequent analysis involved plotting temperature–impact energy curves for comparative assessment, with Boltzmann function fitting applied to determine ductile-to-brittle transition temperatures. Combined macro-/micro-fractography observations ultimately established correlations between laser scanning strategies, notch orientation (governing loading direction), and the cryogenic impact toughness of SLM-TC4 titanium alloy.

## 3. Results and Analysis of the Charpy Pendulum Impact Test

### 3.1. Consistency Analysis of Impact Energy Data

**Figure 6:**
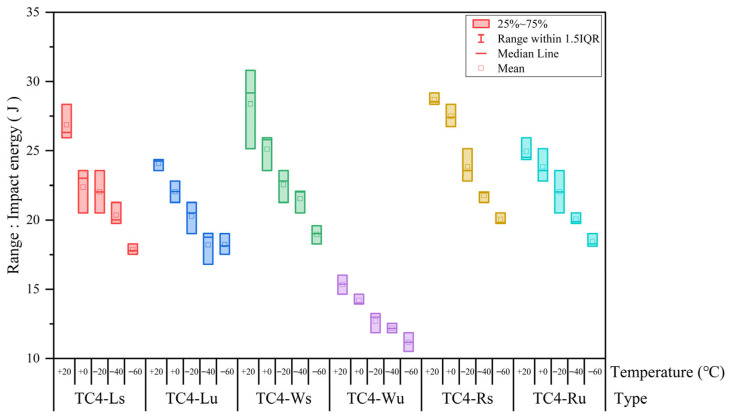
The box plot of impact energy.

The impact energy of alloys comprises two components: energy absorbed during crack initiation and crack propagation stages. For each of the six specimen groups, three replicate tests were performed at every temperature point. To visually assess data consistency across identically processed specimens under varying temperatures, box plots in Figure 6 were generated. These reveal that TC4-Wu maintains excellent consistency across all temperatures, while TC4-Lu, TC4-Rs, and TC4-Ru exhibit moderate consistency, and TC4-Ls/TC4-Ws show relatively low consistency. Notably, all specimens demonstrate improved consistency at −40 °C and −60 °C, attributable to the cryogenic attenuation of texture, porosity, and residual stress effects. TC4-Rs and TC4-Ws exhibit optimal cryogenic consistency, validating their reliability for polar low-temperature applications.

### 3.2. Temperature–Impact Energy Curves

**Figure 7:**
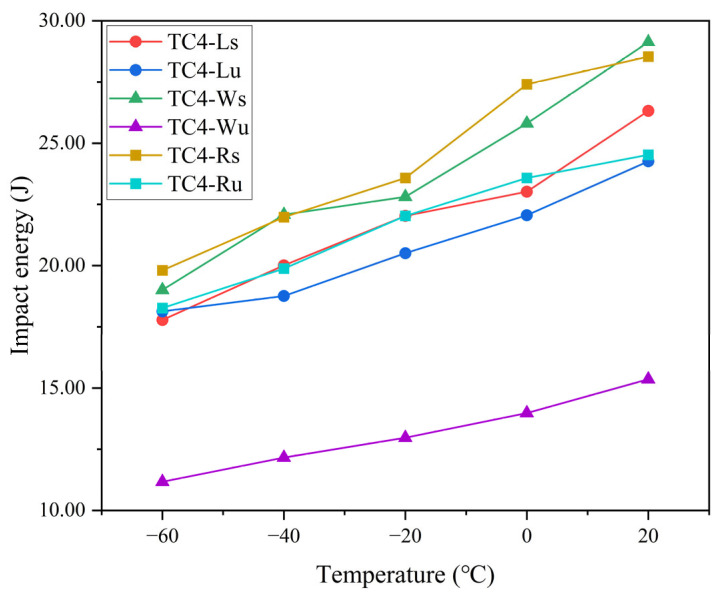
The temperature–impact energy plot.

To mitigate experimental uncertainties from processing and testing, median values of triplicate impact energy measurements at each temperature were selected for analysis (Table 3). The resulting temperature–impact energy plot (Figure 7) demonstrates a consistent reduction in impact energy with decreasing temperature across all specimen groups.

As shown in Table 4, the linear equations corresponding to each group of impact energy and temperature were obtained through fitting. The goodness of fit (*R*^2^) was calculated using Formula (1), and the obtained *R*^2^ values were all greater than 0.90, confirming the positive correlation between impact energy and temperature. Specifically, impact energy at −60 °C decreased by 25–35% relative to 20 °C. Overall impact toughness ranks as: TC4-Rs > TC4-Ws > TC4-Ls ≈ TC4-Ru > TC4-Lu >> TC4-Wu.

(1) $$R^{2}=1-\frac{\sum {\left(E_{actual}-E_{predicted}\right)}^{2}}{\sum {\left(E_{actual}-\bar{E}\right)}^{2}}$$

In the formula, *R*^2^ represents goodness of fit; *E*_actual_ denotes the actual impact energy (J); *E*_predicted_ signifies the predicted impact energy (J); and $\bar{E}$ indicates the average impact energy (J).

**Figure 8:**
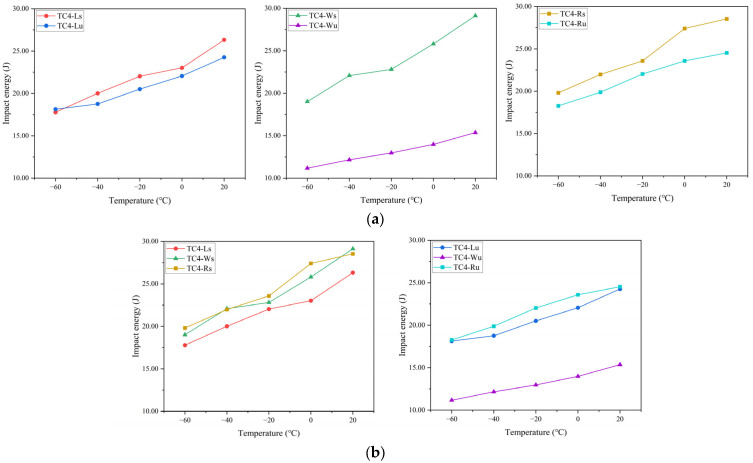
Grouped temperature–impact energy plots: (**a**) by laser scanning strategy; (**b**) by V-notch location.

The six specimen datasets were segregated by laser scanning strategy and V-notch location to generate grouped temperature–impact energy plots (Figure 8). The analysis of Figure 8a reveals that under identical scanning strategies, side-notched specimens consistently exhibit superior impact toughness to top-notched counterparts across test temperatures—except for TC4-Lu, which showed marginally higher impact energy than TC4-Ls by 0.36 J at −60 °C, a difference deemed insignificant within experimental uncertainty. This confirms stronger interfacial bonding strength between deposited layers versus within individual layers at equivalent dimensions. An examination of Figure 8b demonstrates that for identical notch locations, 67°-rotated scanning between layers yields enhanced impact toughness over unidirectional scanning at all temperatures; notwithstanding TC4-Ws exceeding TC4-Rs by 0.59 J and 0.10 J at two temperature points, variations were considered negligible against mean impact energy values. Consequently, TC4-R specimens (regardless of notch orientation) outperform TC4-L/W specimens in impact toughness. Furthermore, within individual layers, TC4-Lu (loaded normal to scanning path) achieves higher impact energy than TC4-Wu (loaded parallel to scanning path), indicating superior toughness perpendicular to the laser scanning direction.

### 3.3. Ductile-to-Brittle Transition Temperature (DBTT)

At room temperature, TC4 titanium alloy primarily consists of an α-phase (hexagonal close-packed, HCP) with a minor β-phase (body-centered cubic, BCC). When test temperatures fall below a critical threshold Tt, the fracture mode transitions from ductile to brittle, termed low-temperature brittle fracture. This transition temperature Tt, defined as the ductile-to-brittle transition temperature (DBTT), serves as a critical indicator of material susceptibility to brittle fracture. It reflects the temperature dependence of material ductility and directly governs the low-temperature service limits by determining toughness retention under cryogenic conditions.

Based on the Charpy pendulum impact test data of metallic materials obtained at the aforementioned series of temperatures, the ductile–brittle transition temperature can be determined. This provides a basis for evaluating the material’s ductile and brittle properties at different temperatures, offering a reference for establishing service conditions for SLM-TC4 components. The relationship curve between impact energy and temperature typically exhibits an S-shaped pattern divided into three characteristic regions: the upper platform region, transition region, and lower platform region. Extensive experimentation and practical experience have demonstrated that the application of the Boltzmann function (as shown in Formula (2) below) to fit and analyze the impact energy versus temperature relationship curve yields statistically significant correlation [22].

(2) $$A_{kv}=\frac{A_{1}-A_{2}}{1+\exp\left[\left(T-T_{t}\right)/\Delta t\right]}+A_{2}$$

In the formula, *A_kv_* represents impact energy (J); *A*_1_ denotes the lower shelf energy (J); *A*_2_ corresponds to the upper shelf energy (J); *T* signifies the test temperature (°C); *T*_t_ is the ductile–brittle transition temperature (°C); and Δ*t* indicates the temperature range of brittle-to-ductile transition (°C). A smaller Δ*t* value reflects a sharper transition process in the material’s ductile-to-brittle behavior.

**Figure 9:**
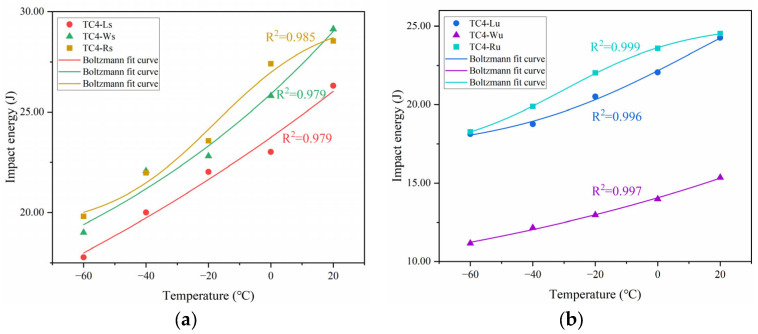
Boltzmann fitting curves and *R*^2^ for impact energy versus temperature: (**a**) TC4-Ls/Ws/Rs; (**b**) TC4-Lu/Wu/Ru.

The experimental data were fitted using the Boltzmann function model in Origin 2024 software, with the results divided into two groups for clarity in visualization (Figure 9), and the corresponding *R*^2^ > 0.95 indicates that the fitted model is valid. The comprehensive analysis of the fitted curves reveals that the curves for TC4-Rs and TC4-Ru exhibit characteristic S-shapes comprising three distinct regions: the lower shelf region, the transition region, and the upper shelf region, corresponding to the material’s brittle fracture zone, ductile–brittle transition zone, and ductile fracture zone, respectively. The ductile–brittle transition temperatures (*T*_t_) for these two specimen groups were determined as −17.8 °C (TC4-Rs) and −30.1 °C (TC4-Ru). In contrast, the datasets for TC4-Ls, TC4-Lu, TC4-Ws, and TC4-Wu failed to exhibit distinct S-shaped characteristics. Consequently, their ductile–brittle transition temperatures could not be ascertained via Boltzmann function fitting, though it is established that these values lie beyond the tested temperature range of −60 °C to 20 °C.

## 4. Fracture Behavior and Microstructural Characteristics

### 4.1. Fractographic Analysis

**Figure 10:**
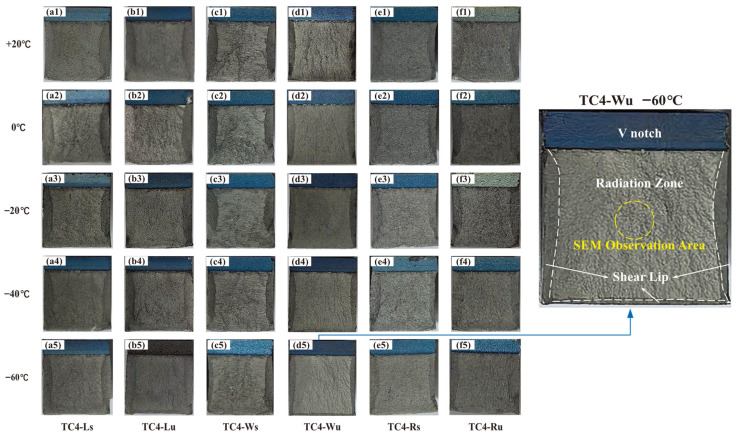
Macroscopic fracture morphology: (**a1**) TC4−Ls, +20 °C; (**a2**) TC4−Ls, 0 °C; (**a3**) TC4−Ls, −20 °C; (**a4**) TC4−Ls, −40 °C; (**a5**) TC4−Ls, −60 °C; (**b1**) TC4−Lu, +20 °C; (**b2**) TC4−Lu, 0 °C; (**b3**) TC4−Lu, −20 °C; (**b4**) TC4−Lu, −40 °C; (**b5**) TC4−Lu, −60 °C; (**c1**) TC4−Ws, +20 °C; (**c2**) TC4−Ws, 0 °C; (**c3**) TC4−Ws, −20 °C; (**c4**) TC4−Ws, −40 °C; (**c5**) TC4−Ws, −60 °C; (**d1**) TC4−Wu, +20 °C; (**d2**) TC4−Wu, 0 °C; (**d3**) TC4−Wu, −20 °C; (**d4**) TC4−Wu, −40 °C; (**d5**) TC4−Wu, −60 °C; (**e1**) TC4−Rs, +20 °C; (**e2**) TC4−Rs, 0 °C; (**e3**) TC4−Rs, −20 °C; (**e4**) TC4−Rs, −40 °C; (**e5**) TC4−Rs, −60 °C; (**f1**) TC4−Ru, +20 °C; (**f2**) TC4−Ru, 0 °C; (**f3**) TC4−Ru, −20 °C; (**f4**) TC4−Ru, −40 °C; (**f5**) TC4−Ru, −60 °C.

Fractography examines the morphology of newly formed surfaces after fracture failure of metallic materials, revealing crack propagation paths, fracture modes, failure types, and underlying causes [23]. Figure 10 displays macro-fractographs of six specimen groups at different temperatures, showing distinct shear lip zones at both lateral edges and the bottom region. This shear lip area diminishes progressively with decreasing temperature. Closer inspection reveals no discernible directional patterns in TC4-Ls (Figure 10(a1–a5)), TC4-Lu (Figure 10(b1–b5)), TC4-Rs (Figure 10(e1–e5)), or TC4-Ru (Figure 10(f1–f5)). Conversely, TC4-Wu (Figure 10(d1–d5)) exhibits faint radial striations propagating from the V-notch tip (see −60 °C inset), while TC4-Ws (Figure 10(c1–c5)) displays pronounced transverse textures. Mechanistically, these transverse textures in TC4-Ws indicate higher crack propagation resistance along deposition directions, whereas the weak radial striations in TC4-Wu suggest easier intralayer crack propagation parallel to the laser scanning path (y-axis). This observation aligns with Section 2.2 conclusions: interfacial bonding strength exceeds intralayer bonding strength, and toughness normal to the scanning direction surpasses that parallel to it. Cross-specimen comparison further confirms TC4-Wu’s smoother fracture surfaces and reduced shear lip areas at identical temperatures, consistent with its inferior impact toughness reflected in experimental data.

**Figure 11:**
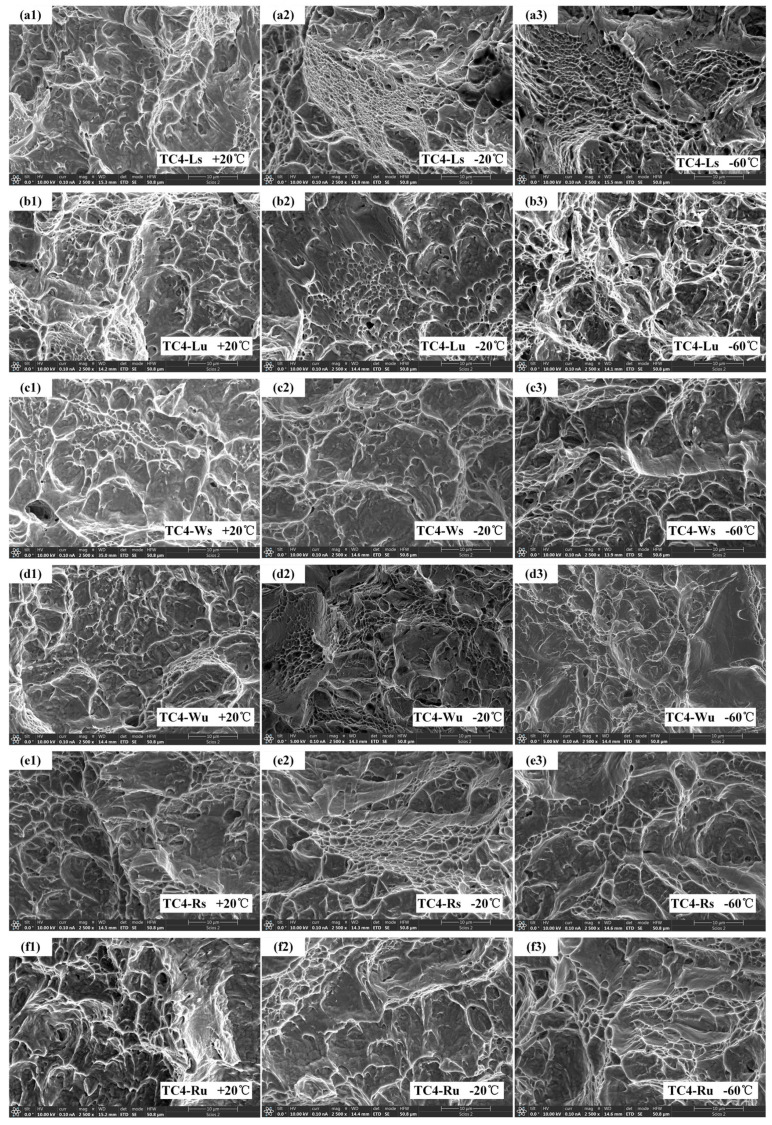
Microscopic fracture morphology: (**a1**) TC4−Ls, +20 °C; (**a2**) TC4−Ls, −20 °C; (**a3**) TC4−Ls, −60 °C; (**b1**) TC4−Lu, +20 °C; (**b2**) TC4−Lu, −20 °C; (**b3**) TC4−Lu, −60 °C; (**c1**) TC4−Ws, +20 °C; (**c2**) TC4−Ws, −20 °C; (**c3**) TC4−Ws, −60 °C; (**d1**) TC4−Wu, +20 °C; (**d2**) TC4−Wu, −20 °C; (**d3**) TC4−Wu, −60 °C; (**e1**) TC4−Rs, +20 °C; (**e2**) TC4−Rs, −20 °C; (**e3**) TC4−Rs, −60 °C; (**f1**) TC4−Ru, +20 °C; (**f2**) TC4−Ru, −20 °C; (**f3**) TC4−Ru, −60 °C.

The macroscopic fracture morphology of six specimen groups was examined at the central region of the fracture zone (highlighted by yellow circles in Figure 10) using scanning electron microscopy (SEM). Figure 11 displays the impact fracture surfaces of distinct specimens at temperatures of 20 °C, −20 °C, and −60 °C, respectively, observed under SEM at a scale of 10 μm.

Fractographic evolution across temperature regimes reveals specimen-specific responses: TC4-Ls exhibits progressively shallower and smaller dimples with decreasing temperature, indicating continuous toughness reduction. TC4-Lu demonstrates diminishing dimples from 20 °C to −20 °C, yet develops uniformly distributed, deeper dimples with pronounced tear ridges at −60 °C—an anomaly attributable to SLM-induced micro-pores nucleating into microvoids under stress, culminating in dimple-dominated fracture. TC4-Ws maintains consistent dimple ridge morphology across temperatures, aligning with macroscopic observations. TC4-Wu displays increased cracking and heterogeneous dimple distribution at −20 °C, correlating with inferior toughness; by −60 °C, distinct cleavage facets emerge in Figure 11(d3) signifying quasi-cleavage fracture, suggesting engagement in the ductile–brittle transition regime. TC4-Rs/Ru specimens show dimple shallowing with deteriorating uniformity from 20 °C to −20 °C, transitioning to prevalent cleavage facets at −60 °C that confirm brittle fracture behavior consistent with Figure 9 Boltzmann fitting.

### 4.2. Microstructural Analysis

**Figure 12:**
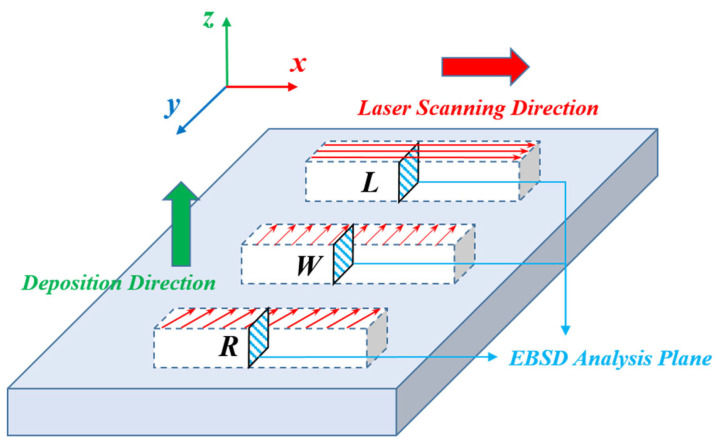
EBSD analysis planes.

To elucidate the underlying mechanisms responsible for the differences in impact toughness among TC4-L, TC4-W, and TC4-R, a systematic EBSD analysis was conducted on all three specimens. The samples were taken from the central region of the YZ cross-section, as shown in Figure 12, which is parallel to the impact fracture surface.

The internal microstructure of SLM-TC4 exhibits an interlaced arrangement with a clearly visible basket-weave morphology, which is composed of acicular marten site. Due to the rapid solidification during the additive manufacturing process, the formation of the β phase is limited, resulting in low β-phase content ranging from 0.51% to 1.09% across the three specimens. The specific α- and β-phase contents are listed in Table 5. In a bimodal microstructure, a higher volume fraction of β phase enhances the material’s deformation capacity under impact loading, enabling greater energy absorption and thus improved impact toughness [24], which is consistent with the higher impact energy value observed in TC4-R.

**Figure 13:**
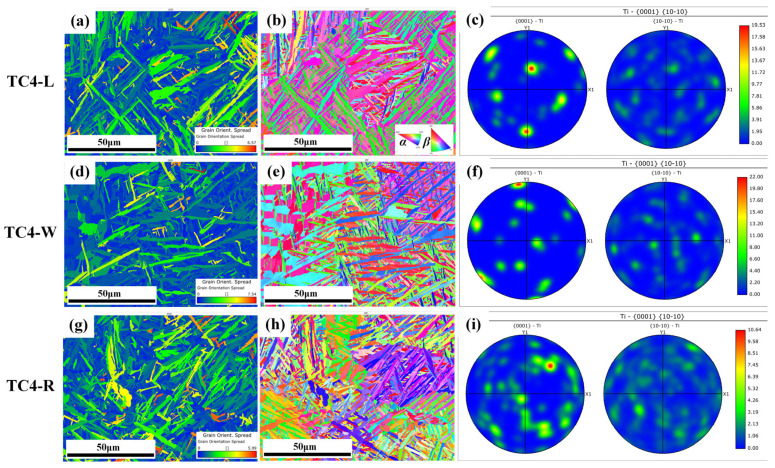
EBSD maps of TC4-L/W/R: (**a**,**d**,**g**) GOS maps; (**b**,**e**,**h**) IPF maps; (**c**,**f**,**i**) α-phase pole figure.

Figure 13 shows the EBSD maps of SLM-TC4. As revealed by the GOS (Grain Orientation Spread) maps, TC4-R exhibits a larger area of yellow and red regions compared to TC4-L and TC4-W, indicating a higher average GOS value. This suggests a higher density of geometrically necessary dislocations accumulated during the layer-by-layer deposition, which facilitates slip-mediated plastic deformation and enhances energy dissipation under impact loading. Furthermore, the IPF (Inverse Pole Figure) maps show that TC4-R displays a more uniform color distribution than TC4-L and TC4-W. This is attributed to the 67°-rotated scanning strategy, which disrupts the preferential grain growth direction and reduces crystallographic anisotropy along the y- and z-axes. In the α-phase pole figure, TC4-R exhibits a more diffused pattern with significantly weakened texture, indicating improved mechanical isotropy within the yz plane. This promotes homogeneous deformation and efficient energy dissipation during impact.

Collectively, these microstructural characteristics—refined basket-weave structure, increased dislocation density, weakened texture, and reduced anisotropy—endow TC4-R with enhanced crack propagation resistance and superior plastic deformation capability under impact loading, which is consistent with its higher experimentally measured impact energy.

## 5. Conclusions

This study conducted Charpy pendulum impact tests from −60 °C to 20 °C on selective laser-melted Ti-6Al-4V (SLM-TC4) specimens fabricated with distinct laser scanning strategies—interlayer unidirectional (TC4-L/W) and 67°-rotated scanning between layers (TC4-R)—incorporating varied notch orientations (top-surface: u; side-surface: s). The full nomenclature of the six specimen groups is detailed in Figure 2 and Figure 4a. By integrating impact energy data with macro-/micro-fractographic analysis, key findings have been established as follows:

(1) Impact energy data consistency universally improves under cryogenic conditions (−40 °C/−60 °C), with side-notched specimens fabricated with 67°-rotated scanning between layers (TC4-Rs) and unidirectional scanning (TC4-Ws), achieving optimal stability, signifying enhanced polar service reliability.

(2) All specimens exhibit significant impact energy reduction (25–35% at −60 °C vs. 20 °C), with toughness ranking: TC4-Rs > TC4-Ws > TC4-Ls ≈ TC4-Ru > TC4-Lu >> TC4-Wu.

(3) The 67°-rotated scanning strategy between layers (TC4-R) increases the impact energy by 7.8% (relative to TC4-L) and 33.7% (relative to TC4-W) on average compared to unidirectional scanning (TC4-L/W) across the tested temperature range. For identical scanning strategies, side-notched specimens (loading direction normal to the deposition plane) exhibit 5.0% (TC4-L), 80.1% (TC4-W), and 11.7% (TC4-R) higher impact energy on average than top-notched specimens (loading parallel to the deposition plane), validating superior interfacial bonding strength between deposited layers versus within layers. Within individual layers, toughness normal to the laser scan path exceeds parallel direction, enhancing design flexibility for load-aligned components.

(4) Boltzmann fitting identifies ductile-to-brittle transitions in TC4-Rs/Ru (Tt ≈ −17.8 °C/−30.1 °C), corroborated by cleavage facets (linked to SLM-inherent porosity) at −60 °C in TC4-Rs, TC4-Ru, and TC4-Wu, indicating shared fracture risks in polar cryogenic applications. Future work should pursue sub −80 °C testing, SLM parameter optimization (e.g., porosity reduction), and the multiphysics coupling analysis of ice load–cryogenic corrosion effects.

## Figures and Tables

**Table 1:** Chemical composition of TC4 (mass fraction, %).

Al	Fe	O	C	V	H	N	Ti
6.05	0.15	0.12	0.03	3.90	0.002	0.005	bal.

**Table 2:** Typical SLM processing parameters.

Laser Scanning Strategy	Laser Power /W	Scan Speed /(mm·s^−1^)	Hatch Spacing /μm	Layer Thickness /μm
Interlayer unidirectional (along x/y-axis)	300	1200	100	61
67°-rotated scanning between layers	300	1200	100	61

**Table 3:** Median impact energy values (J).

Group	20 °C	0 °C	−20 °C	−40 °C	−60 °C
TC4-Ls	26.32	23.02	22.03	20.01	17.78
TC4-Lu	24.26	22.06	20.51	18.76	18.13
TC4-Ws	29.13	25.81	22.81	22.08	19.01
TC4-Wu	15.36	13.98	12.97	12.16	11.17
TC4-Rs	28.54	27.41	23.58	21.98	19.81
TC4-Ru	24.53	23.58	22.03	19.88	18.27

**Table 4:** Impact energy–temperature linear fitting equation and goodness of fit (*R*^2^).

Group	Linear Fitting Equation (E = kT + b) *	*R* ^2^
TC4-Ls	E = 0.183T + 26.4	0.965
TC4-Lu	E = 0.128T + 24.3	0.932
TC4-Ws	E = 0.208T + 28.6	0.973
TC4-Wu	E = 0.088T + 15.3	0.985
TC4-Rs	E = 0.184T + 28.4	0.975
TC4-Ru	E = 0.120T + 24.7	0.915

* E is the impact energy (J), T is the test temperature (°C), k is the temperature coefficient (J/°C), and b is the intercept (J).

**Table 5:** Phase distribution of SLM-TC4 (%).

Phase Name	TC4-L	TC4-W	TC4-R
α phase	96.91	97.46	96.75
β phase	0.88	0.51	1.09
Unknown phase	2.21	2.03	2.16

## Data Availability

The original contributions presented in this study are included in the article. Further inquiries can be directed to the corresponding authors.
